# Empathy and citizen’s social-political attitude: age as a moderator

**DOI:** 10.3389/fpsyg.2025.1663652

**Published:** 2026-01-12

**Authors:** Yingjuan Liu, Lin Lu, Hossein Kaviani, Qingxiang Bao, Junjie Li

**Affiliations:** 1 School of Public Administration, Guizhou University of Finance and Economics, Guiyang, China; 2 University of International Business and Economics, Beijing, China; 3 University of East London, London, United Kingdom

**Keywords:** age moderation, empathy, generational differences, political attitudes, political psychology

## Abstract

**Introduction:**

Building upon prior research linking empathy to political attitudes, this study examines whether age moderates the relationship between empathy and democratic political attitudes—an area that remains underexplored.

**Methods:**

The sample comprised 400 Chinese university students (aged 18–25) and 333 older adults (aged 45–60). Participants completed validated measures assessing empathy and support for democracy, with analyses controlling for normative identity style and demographic factors.

**Results:**

Results indicated that empathy was positively associated with democratic attitudes. Importantly, after controlling for covariates, this positive association remained statistically significant only in the younger cohort, but not among older adults.

**Discussion:**

These findings highlight age-dependent mechanisms in political psychology, suggesting that the drivers of political attitudes differ across generations. The results offer empirical insights for developing political leadership frameworks sensitive to China’s unique sociopolitical environment.

## Introduction

A growing body of research has demonstrated that individuals’ psychological characteristics significantly influence their political attitudes. Traits such as empathy, normative identity style, and egalitarian views on gender roles have been consistently linked to political orientation (Blais and St-Vincent, 2011; Miklikowska, 2012; Mondak and Halperin, 2008; Vecchione and Caprara, 2009). Moreover, these traits tend to vary systematically across demographic groups, with notable age and gender differences. For instance, in the Chinese context, women have been found to show stronger support for egalitarian values compared to men, while younger individuals tend to exhibit higher levels of empathy and more progressive views on gender roles. Conversely, older individuals are more likely to endorse a normative identity style, which emphasizes conformity and tradition (Liu et al., 2018). Similar patterns have been observed in other cultural contexts, including Iran (Kaviani and Kinman, 2017), suggesting a broader cross- cultural relevance.

These individual differences in psychological traits are often shaped by developmental and contextual factors such as parenting practices, educational experiences, and socio-cultural transformations over time (Chang et al., 2011). Understanding how such factors influence political attitudes provides valuable insight into the psychological foundations of political behavior. Among these factors, age has emerged as a particularly important demographic variable, (Rekker et al., 2015), and it also embodies different historical experiences and socialization processes.

Studying the moderating role of age can help us understand the “life cycle effect” and “generational effect” in the formation of political attitudes (Niemi and Klingler, 2012), thereby enriching the theoretical models of political psychology. In the context of China, it can be pointed out that understanding the psychological mechanisms behind the formation of political attitudes across different generations, such as the older generation, which has experienced rapid social transformation, and the younger generation, which has grown up in a globalized and information-rich environment, would hold significant implications for fostering intergenerational communication and designing more inclusive civic education or political communication strategies.

However, the interplay between age and psychological traits, especially empathy, a trait central to both social understanding and political decision-making, has received relatively limited empirical attention. Similarly, normative identity style, which is theoretically and empirically linked to empathy, has been identified as a relevant predictor of political orientation (Pratto et al., 1994), yet its interaction with age remains underexplored.

While prior studies suggest that individuals with higher empathy are more likely to endorse democratic political attitudes, it is not yet clear whether this relationship holds consistently across age groups. Given the rapid socio-economic and political changes experienced in societies like China, significant intergenerational differences in political outlook may be expected. The present study seeks to address this gap by examining whether age moderates the relationship between empathy and support for democratic political attitudes. By exploring this interaction within the Chinese context, we aim to contribute to a more nuanced understanding of how psychological traits interact with demographic variables to shape political behavior across the lifespan.

## Literature review and hypothesis development

### Empathy and political attitudes

Empathy, a fundamental psychological trait, refers to the capacity to comprehend and share the feelings and experiences of others, either through direct perception or imaginative engagement (Telle and Pfister, 2012). It encompasses the tendency to emotionally resonate with others’ affective states and is widely regarded as a precursor to sympathy and compassion. By enhancing emotional understanding and interpersonal connection, empathy plays a critical role in promoting healthy and constructive social relationships (Bailey et al., 2008).

Beyond genetic predispositions, the development of empathy is strongly influenced by environmental factors, including parenting practices, formal education, and peer interactions (Davidov et al., 2013; Durlak et al., 2011; Van der Graaff et al., 2018). Among these, parenting, particularly in early childhood, has been found to play a pivotal role. Parental warmth, characterized by affection, responsiveness, and emotional support, has been consistently linked to higher levels of empathy in children (Zhou et al., 2002). These findings underscore the importance of individuals’ developmental environments and broader social contexts in shaping empathic capacities.

In addition to its interpersonal and developmental relevance, empathy has been identified as a significant predictor of political attitudes. By enabling individuals to understand and relate to diverse perspectives and experiences, empathy may foster openness to difference and more inclusive worldviews (Miklikowska, 2012). Empirical studies have shown that higher empathy is associated with greater acceptance of social diversity (Cundiff and Komarraju, 2008) and reduced gender bias, which in turn can enhance support for women’s political rights and social initiatives (Bäckström and Björklund, 2007).

However, the formation of political attitudes is a complex process influenced by multiple psychological dispositions. To better understand the unique role of empathy, it is useful to consider it in relation to other, contrasting traits, one such trait is normative identity style. This identity-processing orientation involves a strong tendency toward social conformity, wherein individuals adopt the values, goals, and expectations of significant others, such as parents or authority figures, without critical self-reflection (Berzonsky et al., 2011). In contrast to empathy, which fosters openness and perspective-taking, a pronounced normative identity style is generally associated with lower openness to new information and less support for democratic values, instead prioritizing security and tradition (Berzonsky and Sullivan, 1992).

Notably, both empathy and normative identity style share a common developmental root in parenting practices, yet they represent distinct pathways. Authoritative parenting, for instance, has been linked to the development of both traits: it may foster empathy through warmth and support, while simultaneously encouraging a normative orientation by reinforcing conventional values (Zhou et al., 2002; Wang et al., 2008). Crucially, these two traits are themselves negatively correlated, with normative identity style being associated with lower levels of emotional flexibility and perspective-taking (Kaviani and Kinman, 2017; Liu et al., 2018). Therefore, by accounting for the influence of normative identity style, we can achieve a more precise estimation of the specific relationship between empathy and political attitudes.

### The role of age

Political attitudes and the willingness to engage in political activity are also shaped by age. However, this discrepancy may be attributed to age-related differences in personality traits themselves, or it could stem from individuals’ socio-cultural backgrounds and upbringing. For example, research has identified age-related variations in both the cognitive and affective components of empathy. While some studies suggest that empathy tends to decline with age, due in part to reduced social engagement and a diminished ability to infer others’ mental states (Khanjani et al., 2015; Pollerhoff et al., 2022), other findings offer a more nuanced picture. Older adults may experience deficits in cognitive empathy, potentially linked to narrowing social networks, but they often maintain or even exhibit higher levels of emotional empathy, particularly within close, emotionally significant relationships (Richter and Kunzmann, 2011). These findings suggest that empathy develops in a multidimensional and age-sensitive manner.

Over the lifespan, individuals’ political orientations may shift in response to changing social roles, experiences, and generational context. Research has shown that older cohorts are more likely to retain political attitudes shaped by early experiences and to adopt more conservative positions (Cutler and Kaufman, 1975; Goerres, 2009). Younger individuals, by contrast, tend to demonstrate greater openness and are more likely to support progressive and democratic ideals, although they may participate less in traditional forms of political activism (Kiisel et al., 2015). In a study of Chinese citizens aged 18 to 72, Harmel and Yeh (2015) found an inverted U-shaped relationship between age and political engagement, suggesting that political passion peaks in middle age but carries distinct cultural meanings across generations. Additionally, political openness, defined as the willingness to question or challenge existing political structures, has been shown to decline with age, indicating that younger individuals may be more receptive to political change (Harmel and Yeh, 2015; Mondak and Halperin, 2008).

### The present study and hypotheses

Taken together, these findings suggest that age may function as a moderator in the relationship between psychological traits and political attitudes. While prior research has investigated the independent effects of empathy, normative identity style, and age on political orientations, relatively little is known about how these variables interact, particularly in rapidly changing societies like China. Over recent decades, China has undergone significant social, economic, and policy transformations that may have led to pronounced intergenerational differences in political values. Understanding how age moderates the relationship between psychological traits and political attitudes could offer valuable insights into the formation of democratic orientations across different age groups. The present study seeks to address this gap by examining whether age moderates the relationship between empathy and support for democratic political attitudes in a Chinese population. Based on the theoretical framework and prior empirical findings, the following hypothesis is proposed:

*H1*: Empathy will be positively, while normative identity style will be negatively correlated with support for democracy.

*H2*: The elder group will report lower democratic support than the younger group.

*H3*: Age will moderate the relationship between empathy and democratic political attitudes among Chinese citizens.

## Methods

### Participants and procedure

The target sample size was determined prior to data collection, based on power considerations for moderated multiple regression analysis as informed by prior simulation studies (e.g., Stone-Romero and Anderson, 1994; Aguinis and Stone-Romero, 1997; Aguinis et al., 2001; Shieh, 2009). These studies indicate that sample sizes between approximately 226 and 400 provide high statistical power (0.95–0.99) to detect moderation effects. To ensure robust parameter estimates and smaller standard errors, we aimed to recruit a sample substantially larger than this typical range, targeting a moderate-to-large sample size as recommended in recent methodological work (Lüdtke et al., 2020; Zhang and Wang, 2017). The data collection stopping rule was set to achieve this target, concluding recruitment once we had obtained usable data from over 700 participants, which ensured our final sample (*N* = 733) provided ample power for our analyses.

Data collection was facilitated by two trained academic assistants who distributed paper-based questionnaires to voluntary participants. Prior to distribution, approval was obtained from relevant institutional gatekeepers. The assistants provided participants with an overview of the study’s purpose and clearly explained their rights, including anonymity, confidentiality, and the option to withdraw at any time without penalty. Written informed consent was obtained from all participants prior to participation. University students completed the questionnaires in their lecture halls under supervised conditions, while older participants completed them in quiet settings at their workplaces, ensuring minimal distractions and privacy during the process.

733 participants recruited from Guizhou Province, China, ranging in age from 18 to 60 years. Participants were divided into two age groups: the younger group (ages 18–25; M = 21.56, SD = 1.23) and the older group (ages 45–60; M = 51.04, SD = 5.12). The sample consisted of 276 males (37.65%) and 457 females (62.35%). Regarding employment status, 54.30% of participants were university students, 28.10% were office workers, 12.28% were retired, and 5.32% reported other occupations. In terms of educational attainment, 18.28% held a junior or high school diploma, 78.44% had completed an undergraduate degree, and 1.78% held a postgraduate qualification.

### Measures

#### Toronto Empathy Questionnaire (TEQ)

Empathy was assessed using a 10-item subscale of TEQ, which measures an individual’s capacity to understand and respond to the emotional states of others (Spreng et al., 2009). Items were rated on a 5- point Likert scale ranging from 1 (never) to 5 (always), with higher scores indicating greater levels of empathy. A sample item is: “*It upset me to see someone being treated disrespectfully.*” The TEQ is originally composed of 16 items; however, this study employed the validated 10- item version.

#### Normative identity style

Normative identity style was measured using a 7-item subscale from the Normative Identity Style component of the Identity Style Inventory-4 (ISI-4; Smits et al., 2008). This scale evaluates the extent to which individuals adopt goals and standards from significant others without active exploration. Responses were recorded on a 5-point scale from 1 (not at all like me) to 5 (very much like me), with higher scores reflecting stronger normative identity orientation. A sample item is: “*I never question what I want to do with my life, because I tend to follow what important people expect me to do.*”

#### Support for democracy

Support for democracy were assessed using a 9-item subscale from the Support for Democratic Values Scale from support for democracy (SDVS) (Miklikowska, 2012). This instrument measures the degree to which participants endorse democratic principles such as freedom of expression and equality. Items were rated on a 4-point Likert scale from 1 (strongly disagree) to 4 (strongly agree), with higher scores indicating stronger support for democratic values. A sample item is: “*It is necessary that everyone, regardless of their views, can express themselves freely.*”

## Results

The dataset generated and analyzed during this study has been deposited in the Zenodo repository and is publicly available. It can be accessed and cited using the following permanent Digital Object Identifier (DOI): 10.5281/zenodo.17796916. In this study, the descriptive statistics, Pearson correlation analysis, and regression analysis were conducted using the R version 4.2.2. Moreover, hierarchical multiple regression analyses were conducted using a four-step model to test the contribution of covariates, empathy and age groups (main effects), as well as the interaction term (empathy × age group) in predicting social political attitude.

### Descriptive analysis

The descriptive statistical results are shown in Table 1. The mean and standard deviation of empathy, NIS, and democracy were 26.74 (4.23), 21.40 (4.78), and 25.37 (2.82), respectively. In addition, since the absolute skewness value of each scale was less than 2 and the absolute kurtosis value was less than 7 (Finney and DiStefano, 2006), it means that the data were approximately normally distributed.

**Table 1:** Descriptive analysis.

Variables	Mean	SD	Skewness	Kurtosis
Emp	26.735	4.232	−0.141	0.104
Nis	21.402	4.781	−0.025	0.642
Democ	25.372	2.818	0.267	0.285
Young	21.558	1.233	0.326	−0.008
Elder	51.066	5.118	0.444	−1.165

Emp, Empathy; Nis, Normative Identity Style; Democ, Democracy.

### Correlation analysis

The empathy was significantly positively related with NIS (r = 0.124, *p* < 0.001) and democracy (r = 0.111, *p* < 0.01). In addition, NIS was significantly negatively related with democracy (r = −0.129, *p* < 0.001) (see Table 2).

**Table 2:** Correlation coefficients matrix between variables.

Variables	Emp	NIS	Democ
Emp	-		
Nis	0.124***	-	
Democ	0.111**	−0.129***	-

Emp, Empathy; Nis, Normative Identity Style; Democ, Democracy. *p < 0.05; **p < 0.01; ***p < 0.001.

### Hierarchical regression

The hierarchical multiple regression predicting democracy was conducted (see Table 3). In step one of the model, empathy was positively associated with democracy (B = 0.074, *p* < 0.01, 95% CI = [0.026, 0.122]). In step two of the model, empathy remained significant, and the added main effects of NIS (B = −0.069, *p* < 0.01, 95% CI = [−0.112, −0.027]) and undergraduate diploma (B = 1.264, *p* < 0.001, 95% CI = [0.739, 1.788]) both significantly predicted the democracy. However, the difference between people who were retired and those with a junior or high school diploma was not significant, holding other variables constant. In step three of the model, the effects of empathy and NIS on democracy remained significant, and the added main effects of gender (coded as 1 = male and 0 = female; B = −0.409, *p* < 0.05, 95% CI = [−0.811, −0.008]) and age group (coded as 1 = elder group and 0 = younger group; B = −1.536, *p* < 0.001, 95% CI = [−2.019, − 1.053]) were both negatively related to democracy. In step four of the model, the interaction term (empathy × age group) was entered. In this model, the effect of empathy, NIS, and gender remains significant. In addition, the interaction term was significantly associated with democracy (B = −0.118, *p* < 0.05, 95% CI = [−0.209, −0.026]). Specifically, for elder groups, we expect the relationship between empathy and democracy to be 0.209 points per unit lower than younger groups, holding other variables constant.

**Table 3:** Summary of hierarchical regression analysis predicting democracy.

Regressor	Model 1	Model 2	Model 3	Model 4
Empathy	**0.074** (0.02) [0.026, 0.122]	**0.071** (0.02) [0.024, 0.119]	**0.072** (0.02) [0.026, 0.119]	**0.131** (0.03) [0.066, 0.196]
NIS		**−0.069** (0.02) [−0.112, −0.027]	**−0.043** (0.02) [−0.085, −0.001]	**−0.044** (0.02) [−0.086, −0.003]
Education2		**1.264** (0.27) [0.739, 1.788]	0.248 (0.31) [−0.352, 0.849]	0.326 (0.31) [−0.276, 0.927]
Education3		0.610 (0.80) [−0.955, 2.176]	0.720 (0.77) [−0.800, 2.240]	0.657 (0.77) [−0.858, 2.172]
Education4		0.669 (0.86) [−1.017, 2.356]	0.736 (0.83) [−0.902, 2.373]	0.701 (0.83) [−0.930, 2.333]
Gender1			**−0.409** (0.20) [−0.811, −0.008]	**−0.408** (0.21) [−0.808, −0.008]
Age group1			**−1.536** (0.25) [−2.019, −1.053]	1.659 (1.29) [−0.866, 4.184]
Empathy × age group				**−0.118** (0.05) [−0.209, −0.026]
Intercept	23.404	23.941	24.996	23.377
SER	2.802	2.738	2.658	2.648
R^2^	0.012	0.062	0.119	0.127
Adjusted R^2^	0.011	0.056	0.110	0.117

Variables with p values < 0.05 denoted with bold font.

### Moderated effect

**Figure 1:**
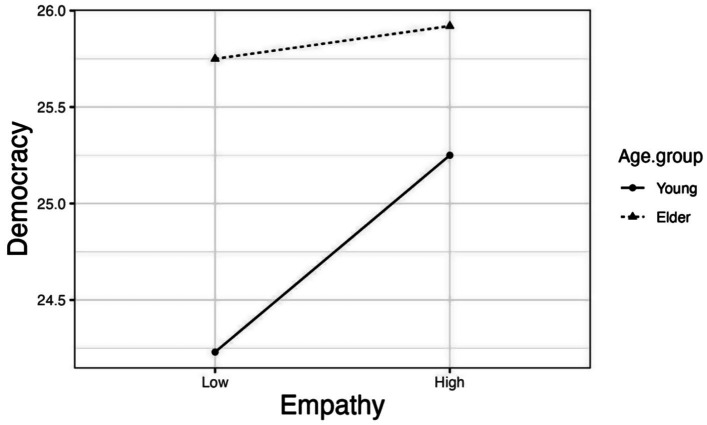
The moderating effect of difference age group.

To further explore the moderating role of age group, we then separated the data by different age group and calculated simple slope of empathy. According to the simple slope analysis, the effect of empathy on democracy was not significant in elder group (Bsimple = 0.021, *p* = 0.50, 95% CI = [−0.039, 0.080]). However, among young people, the effect of empathy on democracy was statistically significant (Bsimple = 0.120, p < 0.001, 95% CI = [0.050, 0.190]). More information can be found in Figure 1.

## Discussion

Support for democracy served as the primary dependent variable in this study. In the first phase of analysis, we examined the relationships among empathy, normative identity style, and democratic attitudes. The results revealed that empathy was positively associated with support for democracy, whereas normative identity style demonstrated a negative association with the same outcome.

However, the relationship between empathy and political attitudes is not unequivocally positive. As the emerging research suggests that empathy, particularly when confined to one’s in-group, can exacerbate political polarization and even contribute to democratic backsliding (Simas et al., 2020). This critical perspective highlights that empathy’s political consequences are not uniform but are contingent upon its target and scope. The crucial distinction depends on whether empathic concern is directed toward in-group members alone or can be extended to out-groups. When empathy is parochial, it may indeed reinforce in-group favoritism and bias. In contrast, the generalized empathy measured in this study is conceptually aligned with the capacity to understand and share the feelings of a diverse range of others. This form of empathy is instrumental in mitigating prejudice against out-groups (Finlay and Stephan, 2000) and underpins the principles of inclusion and equality that are fundamental to democratic systems. Therefore, our finding of a positive link is likely in line with out-group-directed dimension of empathy, which functions as a bulwark against the divisive tendencies of partisan polarization and strengthens support for democracy.

Individuals with a normative identity style tend to adopt the values and norms of authority figures or dominant groups without critical reflection. This predisposition is closely linked to conservative and authoritarian orientations, which are often inversely related to democratic values (Dunkel and Decker, 2012; Pratto et al., 1994). Thus, normative identity style may foster resistance to pluralism and openness, hallmarks of democratic ideology.

The findings also indicated that individuals with higher levels of education, females, and younger participants were more likely to support democratic values compared to their respective counterparts, those with lower educational attainment, males, and older individuals. These patterns are consistent with prior research on democratic values in China, which has shown that younger and more highly educated generations express stronger support for democratic principles (Zhao, 2010). These results not only are consistent with broader trends identified in previous studies (Inglehart and Norris, 2000; Kiisel et al., 2015; Sobolewska and Ford, 2022), but can also be interpreted within the specific socio-cultural context of contemporary China, which is further explored below.

Highly educated individuals are more likely to endorse egalitarian values, which in turn contribute to greater support for democracy and lower levels of authoritarianism (Shu, 2004). For these individuals, higher education may enhance access to diverse sources of information and promote the development of critical and creative thinking, that is, cognitive skills that are strongly linked to democratic orientations (Shu, 2004; Onsman and Cameron, 2014). Moreover, recent research has emphasized that education is positively associated with political engagement (Persson, 2022). Beyond fostering openness and tolerance, knowledge empowers citizens to participate more effectively in political processes, a finding supported across different national contexts (Bovens and Wille, 2021). This trend is also reflected in China, where an increasing number of highly educated young people are becoming involved in political activities. Their greater exposure to global democratic discourses, combined with enhanced analytical capacities developed through education, may contribute to a stronger endorsement of democratic values and a growing desire for political participation.

The higher levels of democratic orientation observed among females and younger participants may be partially explained by their elevated levels of empathy. Prior research has consistently shown that women tend to report higher empathy than men, a difference often attributed to traditional caregiving roles that emphasize emotional attunement and concern for others (O'Brien et al., 2012). This caregiving experience may contribute to the development of stronger empathic capacities. Additionally, women are generally more emotionally responsive and socially attuned, which enhances their emotional empathy and motivation to consider others’ perspectives, especially in social and interpersonal contexts (Huang and Su, 2014; Lennon and Eisenberg, 1987). These traits, in turn, may foster a greater concern for broader social issues and a stronger inclination toward democratic values. Conversely, empathy tends to decline with age, a trend that has been linked to factors such as increased life stress, diminished social engagement, and reduced cognitive-emotional flexibility (Bailey et al., 2008; Grühn et al., 2008; Khanjani et al., 2015; O'Brien et al., 2012). This reduction in empathy among older adults may partially account for their comparatively lower support for democratic values. Importantly, generational differences in openness to new ideas and tolerance of divergent perspectives also play a role. Younger individuals tend to show greater acceptance of novel and diverse viewpoints, a disposition that closely aligns with the core principles of democracy (Huttunen and Saikkonen, 2023). Taken together, and in light of the observed positive association between empathy and democratic attitudes, it is reasonable to infer that the higher levels of empathy typically found in women and younger populations may contribute to their stronger endorsement of democratic values.

An important finding of this study is that age moderates the relationship between empathy and support for democratic attitudes and behaviors. Specifically, after controlling for relevant covariates, the positive effect of empathy on democratic orientation was statistically significant only among the younger group, whereas it was not significant among the older group. Empathy is widely recognized as fostering understanding, tolerance, willingness to cooperate, and conflict resolution skills—all of which are fundamental to the functioning of democratic societies. Young people are in a critical period of value formation and political socialization (Arnett, 2000; Sears and Levy, 2003), during which their comparatively higher levels of empathy may be more effectively internalized as democratic norms, such as openness and egalitarianism, and may promote positive attitudes toward participatory democratic practices (Batson, 2011). As a socio-emotional capacity, empathy may more directly influence young individuals’ identification with democratic principles, including equality, inclusion, and respect for minority rights, as well as their inclination toward civic engagement (Eisenberg et al., 2009; Miklikowska, 2012, 2018). Furthermore, as primary users of social media, young people’s empathy may increase their awareness of diverse perspectives and the challenges faced by marginalized groups online, thereby shaping their democratic participation and attitudes (Ortiz, 2020; Schradie, 2019; Vaccari et al., 2015). Overall, empathy, as a psychological trait that promotes social connection and understanding, plays a crucial role in shaping young individuals’ political worldviews and patterns of civic engagement.

Older adults’ political attitudes and values tend to be more stable and established, with their support for democracy often grounded in long-held convictions, accumulated life experiences, trust in existing institutions, or pragmatic considerations rather than immediate emotional responses such as empathy (Sears, 1983). Consequently, empathy may no longer serve as a primary influence on their mature political perspectives.

In the context of China, the elder generation has lived through significant historical periods, including rapid economic development and profound social transformations, which have deeply shaped their views on democracy, authority, and social order. These formative experiences may exert a stronger influence on their political outlook than affective traits like empathy (Shi and Lu, 2010). As a result, their understanding and enactment of democratic values are likely to differ substantially from those of younger cohorts. The non-significant relationship between empathy and democratic attitudes observed in the older group may thus reflect the characteristics of a mature phase in political attitude development. For elders, democratic stances are often rooted in a stable value system formed through extensive life experience and adaptation to specific socio- political environments (Mishler and Rose, 2001). Core determinants of their democratic attitudes and behaviours may have shifted from socio-emotional factors toward deeper ideological convictions, evaluations of institutional effectiveness, or pragmatic considerations based on personal and collective experience (Goerres, 2009). Moreover, distinct historical contexts have shaped the elder generation’s unique perspective on democracy, the formation of which likely follows mechanisms different from the emotionally driven pathways that characterize younger individuals’ political socialization (Alwin and Krosnick, 1991).

## Limitations

This study has several limitations. First, the cross-sectional design means that all findings are correlational in nature, precluding causal inferences, future research should employ longitudinal or experimental designs to verify the direction of causality. Second, while the study identified the moderating role of age in the relationship between empathy and democratic attitudes, it did not deeply explore the underlying psychological mechanisms. Future work could investigate potential mediators such as “openness to diversity,” “critical thinking,” or “media usage habits” to elucidate these pathways. Finally, the exclusive recruitment of participants from Guizhou Province may limit the generalizability of the results to the broader Chinese population, and expanding the sampling range in future studies would enhance external validity.

## Conclusion

The relationship between empathy and age is a dynamic and evolving phenomenon, explored across multiple disciplines including psychology and neuroscience. Prior research has shown that emotional experiences, such as anger, sadness, and happiness, vary in intensity and relevance across age groups. For example, older women have been found to report emotional congruence comparable to younger women but exhibit greater sympathy overall (Wieck and Kunzmann, 2015). Consistent with these findings, the present study observed that the older group demonstrated higher levels of empathy compared to their younger counterparts. Empathic abilities typically improve throughout adolescence and early adulthood, driven by the maturation of cognitive processes and diverse social experiences. Individuals with higher empathy tend to be more accepting of diversity, better able to understand and share the feelings of others, and more open to alternative perspectives and experiences. Consequently, empathy is positively associated with democratic political attitudes. Importantly, this study identified a significant interaction between age and empathy in shaping political attitudes. Among older adults, empathy did not significantly predict democratic attitudes, likely due to the stability of their political beliefs formed over decades of life experience. In contrast, among younger individuals, higher empathy significantly and positively predicted support for democracy. These findings suggest that empathy plays a more direct role in shaping democratic orientations during early political socialization, whereas other factors may predominate in later adulthood.

## Data Availability

The datasets presented in this study can be found in online repositories. The names of the repository/repositories and accession number(s) can be found in the article/supplementary material.
